# Acoustic shock waves, a new weapon against angina?

**DOI:** 10.1007/s12471-016-0829-3

**Published:** 2016-04-07

**Authors:** N. M. van Hemel, J. F. Verzijlbergen

**Affiliations:** Department of Cardiology, University Medical Center , Utrecht, The Netherlands; Department of Nuclear Medicine, Erasmus MC, Rotterdam, The Netherlands

In the years before direct revascularisation for drug-refractory angina pectoris, some indirect methods were explored to improve myocardial ischaemia. Carotid nerve stimulation activated by the patient when angina emerged and Vineberg surgery [1] with implantation of the free end of the mammary artery (started about 1963) are examples of desperate attempts to suppress untreatable symptoms. Both were abandoned because of non-proven effects in contrast to direct revascularisation, which arrived around 1967. The favourable effects and safety of the current percutaneous coronary intervention and bypass surgery permit direct revascularisation in almost all categories of patients with myocardial ischaemia. However, for some patients direct revascularisation or redo procedures are not feasible because of the poor condition of the coronary arteries, age or major comorbidity; the number of these patients is gradually increasing. This observation initiates new experimental, non-invasive methods to improve coronary circulation and myocardial perfusion with the option of relief of untreatable angina. These attempts vary from nerve stimulation to gene therapy [2].

Acoustic pulse waves are used in an experimental attempt to eradicate drug-refractory angina because it initiates angiogenesis in experimental conditions [3]. With an amount of energy about 10 % of that usually applied to crush kidney stones, several favourable bio-effects have been documented. An increase of vascular endothelial growth factor and of capillary density resulting in a better left ventricular ejection fraction, wall thickening fraction and myocardial blood flow of the ‘treated’ area could be documented. Several preliminary studies in patients with severe angina pectoris without other therapeutic options could show promising results in qualitative terms. However, as Slikkeveer and coworkers [4] stated, quantitative observations of changes of myocardial blood flow in the target ischaemic area after application of acoustic pulse waves have not been published until now.

In their small pilot study of patients with severe angina pectoris and major coronary disease, these authors could not observe an obvious improvement of the blood flow four weeks after the last application of acoustic pulse waves by measuring myocardial blood flow with cardiac magnetic resonance imaging (CMR) [5]. Nevertheless, despite an unchanged frequency of angina attacks, some improvement in the physical condition and less use of nitroglycerin were noted. The authors concluded that this negative outcome requires more scientific insight into the bio-effects of acoustic pulse waves before further clinical exploration can be justified.

With full appreciation for the efforts of the investigators, some elements of this clinical pilot study that might affect the reported results should be pointed out. The course of patient symptoms, timing and methods of measurement of myocardial blood flow after acoustic pulse waves, and ‘collateral damage’ of tissue beyond the target zone are questionable. The unchanged frequency of attacks conflicts with a better perceived physical condition according to New York Heart Association class, and less daily use of nitroglycerin, respectively. Despite use of a disease-specific quality of life questionnaire, one can safely assume that the small cohort of patients, as well as a placebo effect resulting from frequent and intensive attention and care of the study patients, affect the documented changes of health perception and symptoms. Therefore this part of the outcome of the pilot study can be classified as arbitrary.

The follow-up time after application of the acoustic pulse waves needs to be questioned. At three months after the last application, quality of life was again determined whereas the time of the second CMR was not precisely presented, presumably in conjunction with filling out the questionnaire, in total about 5 months after the last application. This interval appears too short to permit a reliable quantification of new angiogenesis and presumably other bio-effects. In the past, at least 6 months of follow-up after Vineberg surgery was required to determine any contribution of the implanted artery to myocardial perfusion. A longer follow-up and an additional CMT, e. g. after a further six months, could have shown a different picture.

The acoustic pulse waves were applied towards previously mapped ischaemic zones. To catch the entire zone, the application area was extended including ‘treatment of healthy segments’ [4]. This approach can elicit negative effects because the bio-effects on ‘normal’ human myocardial tissue with a fixed dosage are uncertain. More importantly, the so-called ischaemic zone in the end stage of coronary heart disease is a conceptual area rather than a well-described entity. Pathology of these hearts shows unclear border zones intermingled with fibrosis, scar tissue and surviving myocytes [6]. That mixture excludes a sharp anatomical separation of the target zone and healthy neighbours without ischaemia and complicates the direction and penetration of the ‘beam’ of the acoustic pulse waves. One can consider whether application of pulsed acoustic waves in this specific patient group brought more harm as possible ‘collateral damage’ of tissue beyond the target area than benefit.

CMR is well established for assessing myocardial volumes and function, and for myocardial fibrosis and (non)ischaemic heart diseases. In regions with reduced perfusion, concentrations of Gadolinium are decreased, resulting in lower signal intensity. Recently, different techniques have been developed to improve quantification, aiming to determine a reliable tissue function. None of these techniques were employed in this study. The accuracy of the employed quantification technique is insufficient to calculate absolute flow in these difficult areas [7].

We agree with the authors that our knowledge of the bio-effects of pulsed acoustic wave shocks applied to specific myocardial targets is so limited that more knowledge is strongly needed. Consequently one cannot explore this ‘treatment’ in new patients before we know more about the precise individual dosages, the optimal timing and the best method to measure positive and side effects of acoustic pulse waves. Until now, this indirect treatment has not proven to constitute a beneficial and harmless “weapon” against drug-refractory angina. Whether other treatment indications in cardiology can profit from acoustic pulse waves, also remains questionable.
